# Multiple Primary Melanoma Associated with CDKN2A Mutation—Case Report and Review of the Literature

**DOI:** 10.3390/medicina60050763

**Published:** 2024-05-05

**Authors:** Luana-Andreea Nurla, Mariana Aşchie, Georgeta Camelia Cozaru, Mădălina Boșoteanu

**Affiliations:** 1 Department of Dermatovenerology, “Elias” Emergency University Hospital, 011461 Bucharest, Romania; 2 Institute of Doctoral Studies, Doctoral School of Medicine, “Ovidius” University of Constanţa, 90052 Constanţa, Romania; 3 Clinical Service of Pathology, “Sf. Apostol Andrei” Emergency County Hospital, 011461 Constanţa, Romania; 4 Department of Pathology, Faculty of Medicine, “Ovidius” University of Constanţa, 90052 Constanţa, Romania; 5 Department VIII—Medical Sciences, Academy of Romanian Scientists, 050044 Bucharest, Romania; 6 Center for Research and Development of The Morphological and Genetic Studies of Malignant Pathology (CEDMOG), 900591 Constanţa, Romania

**Keywords:** multiple primary melanoma, CDKN2A mutation, immuno-histochemistry, case report, literature review

## Abstract

The CDKN2A gene remains understudied in melanoma compared to BRAF alterations. Inactivation of this tumor suppressor gene through homozygous deletions in the 9p21 chromosomal region leads to cellular proliferation and disrupts pro-apoptotic pathways. Genetic changes in CDKN2A are linked to multiple primary melanomas (MPM), with patients diagnosed with melanoma facing an elevated risk of developing additional primaries. We present the rare case of a 72-year-old Caucasian woman with nine metastasizing melanomas across diverse anatomical sites, posing a diagnostic challenge. Initial diagnosis in 2022 revealed ulcerated superficial spreading melanomas, progressing to intradermal and papillary dermal populations with neurotropism and angiotropism by early 2023. Lymph node metastases were identified, classifying the condition as pT3b N3b. Subsequent assessments in April 2023 revealed clinically suspicious melanocytic lesions diagnosed as intradermal and traumatized junctional nevi. In late 2023, cutaneous pigmented lesions and subcutaneous metastases were confirmed as nodular nevoid low-CSD multiple melanomas. Fluorescence in situ hybridization testing revealed homozygous CDKN2A deletion, necessitating close multidisciplinary collaboration for an optimized care plan for effective monitoring and intervention in this intricate clinical scenario. In summary, this case report highlights the diagnostic challenges of MPM in a single patient. Stressing the importance of immuno-histochemistry and CDKN2A genetic testing, our findings underscore the crucial role of these tools in accurately distinguishing malignant melanocytic proliferations from nevi and characterizing MPM cases.

## 1. Introduction

Frequently denoted as p16 or INK4a/ARF, mutations in the CDKN2A gene are a less commonly explored aspect within the context of melanoma when compared to BRAF alterations. The CDKN2A gene, functioning as a tumor suppressor, undergoes inactivation through homozygous deletions situated in the 9p21 chromosomal region, which leads to cellular proliferation and disruption of pro-apoptotic pathways. It has been demonstrated that these genetic changes are linked to cases of multiple primary melanomas (MPMs) and the maximum number of primaries reported in a patient was 13, with a mean per individual ranging from 2.1 to 2.8 [1]. Individuals diagnosed with melanoma face an elevated risk of developing additional primary melanomas and the occurrence of MPM is recognized in 0.2 to 8.6% of patients initially diagnosed with a single melanoma, who have been reported to experience an overall poorer survival outcome [2].

The present case report aims to highlight the clinical diagnostic challenges occurring in patients without specific skin-cancer risk factors and to underscore the importance of series surgical excisions performed for multiple suspicious melanocytic lesions.

## 2. Case Presentation

A 72-year-old Caucasian female patient, resident in an urban area with over 200,000 inhabitants and an average annual sunshine of 2299 h (latitude 44°10′50.63′′ N and longitude 28°38′3.55′′ E) presented, in August 2022, in the ambulatory department of the Dermatology Clinic of an academic hospital, for self-observed recent changes in multiple melanocytic lesions on different cutaneous areas, prompting investigation for melanoma.

The hereditary collateral history included essential arterial hypertension and diabetes mellitus diagnosed in her mother and prostatic cancer in her father, with no significant cutaneous pathology detected in any first- or second-degree relative. Moreover, personal comorbidities, such as arterial hypertension and dyslipidemia, were associated; the patient had retired at the time of the first hospital referral, but the anamnesis revealed that her previous job was that of a call center representative and, therefore, did not have professional exposure to ultraviolet radiation. Personal sun-damage risk factors included the Fitzpatrick phototype II and a history of sunburn in childhood. No diagnostic challenges related to access to healthcare, cultural, or financial matters were associated with the analyzed case.

**Figure 1:**
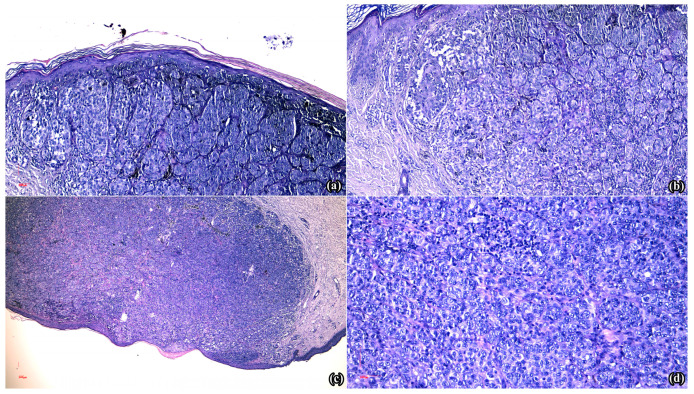
Microscopic aspect of the primary melanomas: (**a**) Atypical epithelioid melanocytic population with nodular, en-dome disposition, Breslow index 1 mm, with epidermal consumption without ulceration and mitotic activity > 1/mm^2^ (lesion from the right thigh, HE × 10); (**b**) Atypical epithelioid melanocytic population with nodular, en-dome disposition, Breslow index 1.5 mm, with epidermal consumption without ulceration and mitotic activity > 1/mm^2^ (lesion from the left foot, HE × 10); (**c**) Atypical epithelioid melanocytic population with massive architecture, Breslow index 2 mm, with pagetoid extension, but without epidermal ulceration (lesion from the left shoulder, HE × 5); (**d**) Atypical epithelioid melanocytic proliferation (lesion from the left shoulder, HE × 20).

The diagnostic approach involved clinical and dermoscopic assessments that revealed Fitzpatrick phototype II, associated with approximately 30 pigmented nevi exhibiting irregular shapes, diverse coloration (ranging from light to dark brown), and varying diameters. Total body mapping (TBM) was deployed to capture photographic documentation of the entire body surface, followed by sequential digital dermoscopy (FotoFinder bodystudio ATBM master system, 4th Generation, FotoFinder Systems GmbH, Bad Birnbach, Germany) of specific melanocytic lesions, with the goal of analyzing their evolution over time, as well as to identify any newly emerging lesions. Elements suggestive of malignancy (such as irregular borders, shiny white streaks, and irregular pigmented network) were identified in four melanocytic lesions, therefore surgical excision was decided. The histopathological assessment of the pigmented macule located on the cutaneous surface of the left mammary gland revealed atypical melanocytic epithelioid non-ulcerated proliferation, measuring 0.4/0.4/0.2 cm, with massive pattern, a Breslow index of 1 mm, mitotic rate > 1/mm^2^, and present vascular invasion. Moreover, the lesion developed on the left shoulder had microscopical characteristics of an atypical melanocytic epithelioid non-ulcerated population, measuring 0.7/0.6/0.2 cm, with massive architecture, a Breslow index of 2 mm, significant pagetoid extension and mitotic rate > 1/mm^2^ (Figure 1c,d). The third excised fragment, originally located in the right lateral cervical region, exhibited an ulcerated proliferation of atypical epithelioid melanocytes of 0.6/0.6/0.15 cm, with massive architecture, a Breslow index of 1.5 mm, present angiotropism and mitotic rate > 1/mm^2^. The last sample obtained from the left calf displayed an atypical pigmented epithelioid ulcerated proliferation of 0.6/0.6/0.21 cm, with massive architecture, a Breslow index of 2.1 mm, angiotropism and mitotic rate > 1/mm^2^. Therefore, the final diagnosis was that of ulcerated superficial spreading melanoma, with multiple localizations (pT3b(m)).

Immuno-histochemical testing was considered appropriate, hence a panel comprising seven antibodies was used (Table 1).

**Figure 2:**
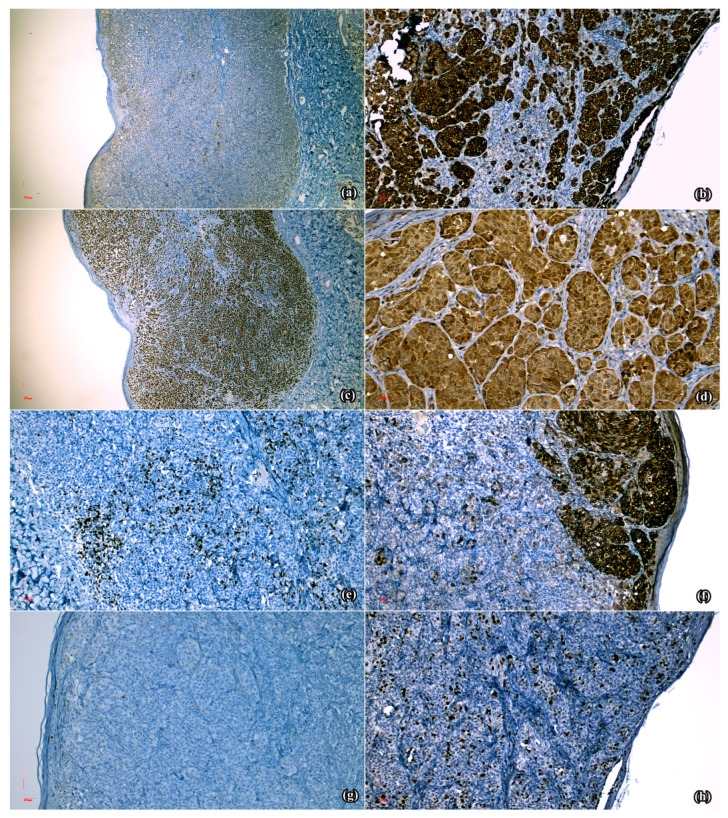
Microscopical aspects of the immuno-histochemical reactions obtained in the analyzed specimens: (**a**) Diffuse positive reaction in the melanocytic population (S100 × 5); (**b**) Diffuse positive reaction in the melanocytic proliferation (MelanA × 10); (**c**) Diffuse positive nuclear reaction in the neoplastic population (SOX10 × 5); (**d**) Present expression in the cytoplasm and sporadically at the nuclear level in the melanocytic population (p16 × 20); (**e**) Positive reaction in the intra- and perilesional lymphocytic infiltrate (CD8 × 10); (**f**) Positive reaction in the intra-epidermal and superficial dermal component, and focal absent reaction in the deep dermal proliferation (HMB45 × 10); (**g**) Positive nuclear reaction in 5% of the melanocytic population (lesion from the left shoulder, Ki-67 × 10); (**h**) Positive nuclear reaction in 25% of the melanocytic population (lesion from the right lateral cervical region, Ki-67 × 10).

S100 (Figure 2a), PAN MELANOMA CK2 (Figure 2b) and SOX-10 (Figure 2c) displayed diffuse positive reactions. P16 had present expression in the cytoplasm and sporadically in the nuclei (Figure 2d), while CD8 revealed positive reaction in the intra- and peritumoral infiltrate (Figure 2e). Moreover, HMB-45 determined positive reaction in the intra-epidermal, superficial dermal areas and focal absent expression in the deep dermal melanocytic population, suggestive of paradoxical maturation (Figure 2f). Finally, Ki-67 generated positive nuclear reactions of variable intensities, ranging from 5% in the tumor on the left shoulder (Figure 2g) to 25% in the right lateral cervical lesion (Figure 2h).

The final diagnosis was melanoma with paradoxical maturation and re-excisions with safety margins of 1 cm for the scars on the left breast and right lateral cervical region, and 2 cm for the tumors on the left shoulder and left calf, respectively, were performed. Clinical dermatological and dermoscopic follow-ups at 3-month intervals were initiated.

In February 2023, the diagnostic process continued with other multiple excisions of cutaneous tumors. From a histological perspective, the scalp tumor revealed a 0.6/0.6/0.6-cm atypical melanocytic epithelioid ulcerated population located intradermally, with focal hypodermic extension, without epidermal ulceration, a Breslow index of 4 mm, present angio- and neurotropism, mitotic rate of 2/mm^2^ and non-brisk inflammatory infiltrate. The lesion excised from the left lateral cervical region displayed an atypical melanocytic epithelioid non-ulcerated population measuring 0.4/0.2 cm, located in the papillary dermis, with epidermal pagetoid ascension, a Breslow index of 1.5 mm, mitotic dermal rate of 2/mm^2^, with angiotropism, intra- and peritumoral brisk inflammatory infiltrate. The pathology report corresponding to the lesion obtained from the mandibular area described an atypical melanocytic epithelioid ulcerated population measuring 0.9/0.6 cm, with epidermal pagetoid ascension and epidermal consumption, a Breslow index of 2 mm, dermal mitotic rate of 3/mm^2^, with angiotropism, regression and non-brisk intra- and peritumoral inflammatory infiltrate. The lymphoid tissue collected from the right mandibular region comprised four lymph nodes with massive melanoma metastases, composed of a tumoral deposit with maximal diameter of 4 mm and extra-capsular extension of 2 mm. The final diagnosis was of multiple low-CSD nodular melanomas with 4/4 lymph node metastases in the right mandibular region with characteristics of epidermotropic cutaneous metastases (pT3b N3b), changing the pTNM staging to stage IIIC.

In April 2023, the evaluation performed in the Dermatology Clinic identified three melanocytic lesions with signs of progression that were excised and histopathologically diagnosed as nevi.

Later, in August 2023, three lesions were supplementarily excised and the microscopical evaluation of the one located on the right thigh (Figure 1a) described an atypical melanocytic epithelioid ulcerated population, measuring 0.5/0.4 cm, with nodular dome-shaped disposition, a Breslow index of 1mm, epidermal consumption without ulceration, mitotic rate of >1/mm^2^, and intra- and peritumoral brisk inflammatory infiltrate. Moreover, the excised nodule from the left foot displayed, at the microscopic examination, an atypical melanocytic epithelioid ulcerated population of 0.4/0.4 cm, with nodular dome-shaped disposition, a Breslow index of 1.5 mm, epidermal consumption lacking ulceration, a mitotic rate of >1/mm^2^, and intra- and peritumoral brisk inflammatory infiltrate (Figure 1b). The mass located on the anterior thorax comprised subcutaneous conjunctive and adipose tissue that revealed microscopic characteristics of an atypical melanocytic epithelioid proliferation with nest-like disposition, moderate pigmentation, tumoral necrosis and mild lymphocytic infiltrate, measuring 1/0.8/0.5 cm. Furthermore, supplementary imagistic tests were performed, consisting of thoracic, abdominal and pelvic CT scans, as well as cerebral MRI, in order to detect potential visceral metastases. However, the results showed no pathological masses or changes in the aforementioned explored territories.

The final diagnosis was nodular nevoid low-CSD multiple melanoma and subcutaneous melanoma metastasis on the anterior thorax (pT2a pM1a), placing the pathological entity in stage IV. Eventually, the patient was diagnosed, up to now, with nine primary cutaneous melanomas (Table 2).

**Figure 3:**
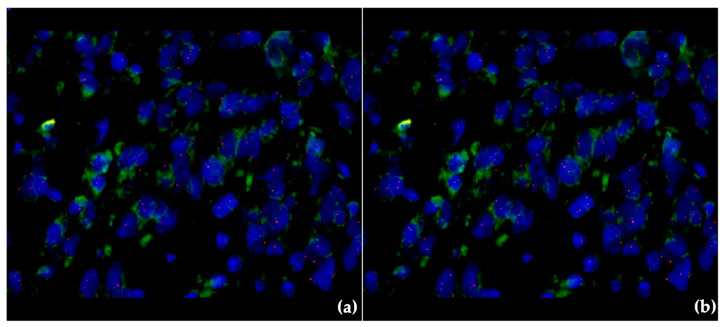
Fluorescence in situ hybridization: (**a**) Homozygous CDKN2A deletion in the melanoma lesion on the right lateral cervical region; (**b**) Homozygous CDKN2A deletion identified in the cutaneous melanoma lesion developed on the right thigh.

Furthermore, BRAF testing was performed for all the diagnosed tumors and revealed negative results, therefore genetic testing for CDKN2A mutation was considered appropriate. The latter investigation was executed at the Center for Research and Development of the Morphological and Genetic Studies of Malignant Pathology (CEDMOG) via dual-color fluorescence in situ hybridization (FISH) performed on formalin-fixed paraffin-embedded material. Slides were evaluated, and images were captured using an epi-fluorescent microscope (Zeiss microscope–Axio Imager.M2, Zeiss, Oberkochen, Baden-Württemberg, Germany) equipped with suitable filters and an image-analysis system (MetaSystem Isis, Reggio Emilia, Italy). In all the samples, we evaluated at least 100 non-overlapped intact interphase nuclei, characterized by uniform 4′,6-diamidino-2-phenylindole (DAPI) staining with intact nuclear contours, of consecutive cells in at least two different areas of the section for each sample. Among them, two tumoral fragments contained ≥15 nuclei that lacked both signals for CDKN2A (no green signal), corroborated with at least one signal for chromosome 9 centromere (a minimum of one orange signal), and therefore these were considered positive for homozygous CDKN2A deletion (Figure 3).

The patient is currently undergoing Pembrolizumab immunotherapy, with optimal tolerability and therapeutic adherence. Future surveillance requirements include dermatological and video-dermoscopic assessments every 3 months, as well as regular oncological check-ups that decide the sequence of imagistic evaluations (comprising lymph node ultrasounds, thoracic, abdominal and pelvic CT scans and cerebral MRIs). Moreover, given the fact that the patient is a carrier of the CDKN2A mutation, she was counseled regarding the risk of familial melanoma and regular cutaneous examinations were recommended for her two daughters.

## 3. Discussion

The particularities of the presented case reside in the high number of primary melanomas detected in a single individual, especially given the fact that no classical risk factors (such as chronic or repeated acute exposure to ultraviolet radiation or familial history of cutaneous melanoma) were associated with their occurrence, as well as the advanced age at the time of the first diagnosis, compared to the reported mean age (58.2 years) of the initial melanoma diagnosis in women [3].

In a study conducted by Ungureanu et al., focusing on Romanian patients, as in our case, it was revealed that a total of 59 melanomas were diagnosed in 26 patients, averaging 2.3 melanomas per individual [4]. Among the cohort, the majority of patients experienced the development of precisely two melanomas, with a maximum number of four melanomas diagnosed in two patients [4], thus highlighting the scarcity of cases with nine primary melanomas in the Romanian population. To our knowledge, the reported case with the highest number of multiple primary melanomas in the literature was revealed in a study by Slingluff et al. in 1993, that discovered a patient with 48 melanomas [5].

In our report, the anatomical distribution of the melanoma lesions did not follow a specific pattern, taking into account the fact that only one tumor was located on the trunk, three were detected on the lower extremities, one on the upper extremities and the other four were identified in the head and neck area. Only one paper focusing on the North Carolina population described the cervical and cephalic areas as the most commonly affected sites [6], while the majority of studies identify the lower extremities as the most frequently involved anatomical sites in women [7].

In a recent study, it was shown that 3.8% of the analyzed individuals experienced the onset of a second primary melanoma during the median follow-up interval of 61 months, but the locations and histopathological subtypes of the initial and subsequent melanomas did not follow a certain consistency pattern [8]. Even though the second melanomas described in the literature had smaller Breslow indexes compared to the initial occurrences [8], our patient exhibited other histological subtypes of secondary melanomas (nodular melanoma), with elevated tumoral thickness. These findings highlighted the particular aggressive tumor’s biological behavior identified in our case, despite the fact that the patient followed a rigorous specialty monitoring plan and cutaneous self-surveillance and did not encounter follow-up challenges leading to the evasion of medical surveillance due to noncompliance or relocation, as has been recorded in other cases [7].

Characteristics, such as fair phototype and a large number of pigmented nevi (>100 in an individual) or of cherry angiomas (>50 per patient), were identified as independent risk factors for the development of the second primary melanoma [8]. The sex of our patient was congruent with the female preponderance described in the literature, especially given the fact that 71% of patients with at least three primary melanomas are women [9,10].

Concerning the particularities encountered in patients with CDKN2A mutations, a study by Taylor et al., investigating the role of the aforementioned genetic alteration on the types and numbers of nevi detected in melanoma-prone families and sporadic cases, found that CDKN2A mutation carriers exhibited a generally greater prevalence of atypical nevi than CDKN2A-wild type patients [11]. Moreover, patients diagnosed with this mutation had an approximately double risk of developing numbers of nevi higher than the median value established per center, compared to wild-type carriers [11]. However, when categorizing individuals based on melanoma case status, more significant positive correlations between CDKN2A pathogenic mutations and nevus counts were noted among family members not affected by melanoma [11].

On the other hand, mutations in the CDKN2A gene are mainly associated with an elevated risk of oncological conditions, such as melanoma and pancreatic cancer [12], entities identified through specific genetic tests in about 38% of families with melanoma history [13]. Nevertheless, only between 3.2% and 15% of patients with multiple primary melanoma lacking familial history are also CDKN2A mutation carriers [13].

In a study conducted by Marghoob et al., an inverse correlation between the total nevus count and cutaneous melanoma thickness was discovered [14]. Their findings demonstrated that individuals with a higher number of nevi tended to have thinner cutaneous melanomas and a higher prevalence of in situ melanomas, regardless of sex and age [11]. However, the presented patient had a mean Breslow index of 1.84 mm, with a minimum of 1 mm and a maximum value of 4 mm, even though the clinical dermatological exam did not reveal an elevated number of nevi. Moreover, in our case, all the examined malignant lesions were de novo melanomas, not nevus-associated melanomas. According to the literature, nevus-associated melanomas are frequently observed in younger patients who have multiple atypical nevi, usually exhibiting the histological subtype of superficial spreading melanoma, which tend to have a lower Breslow thickness and a more favorable prognosis when compared to de novo melanomas [15,16]. On the other hand, a review study emphasized that most nevus-associated melanomas originate from common acquired nevi, with a preference for dermal nevi—a type of nevus that typically forms in early childhood [17].

In patients with MPM, approximately 26–40% of cases were documented to have synchronous lesions, with the remaining instances presenting as asynchronous tumors [18], as compared to the first four melanoma entities that were diagnosed at the same time, during the initial dermatological visit of the presented patient. The elevated risk of developing subsequent melanomas, especially in the first year following the initial melanoma diagnosis and also in the first three years after the first diagnosis (in 49% of patients) [19], was identified in the reported case, underscoring the significance of conducting comprehensive skin examinations for melanoma patients, not only during the initial visit but also during subsequent follow-ups.

Given the fact that cases of multiple primary melanoma do not benefit from specific therapeutic resources other than those destined for the tumor with the greatest Breslow index, and, therefore, with the most advanced stage of disease, periodic full skin examinations and lifelong specialty follow-up exams are crucial for the early detection of a subsequent primary melanoma lesion, as well as for the identification of potential metastases [1]. Wide local surgical excision of any second or consequent primary melanoma is the mandatory step required for diagnostic confirmation and staging and it may be performed in a safer manner and with better survival outcomes inversely proportional to the tumoral thickness [20]. The data obtained from a research conducted by Ni et al. showed that the number of primary melanomas does not represent an independent risk factor concerning mortality rates, and that the most important favorable predictive parameter is defined by low Breslow index values in patients with at least one cutaneous melanoma [21].

A case series including patients with advanced melanomas and one or more other non-melanoma oncological diseases explored the choice of therapeutic strategies employed for the melanocytic tumors, as well as for the non-cutaneous malignancies [22]. Concerning the treatment strategies used for melanoma, the majority of patients (90.9%) underwent surgery, systemic therapy including immunotherapy, targeted therapy and cytotoxic chemotherapy (81.8%) and a minority of them was exposed to radiation therapy (9.1%) [22]. A total of 54.5% of the patients received immune checkpoint inhibitors such as PD-1 and CTLA-4 inhibitors [22] and previous studies demonstrated that, even though the efficacy of these agents is significant on first primary melanomas, their interference with the development of second primary melanocytic malignant tumors or other non-cutaneous cancers remains controversial [22]. The analysis by Heudel et al. revealed a lower risk of MPM in patients treated with immunotherapy for the first primary tumor, compared to those that followed a standard chemotherapy scheme [23]. Due to the fact that immune checkpoint inhibitor (ICI) therapy was approved in 2011 in the US, an investigation based on data available through the Surveillance, Epidemiology, and End Results (SEER) Program compared the outcomes of melanoma patients managed in the pre- (2005–2010) and post-ICI (2011–2016) era and found discrepancies in MPM incidence, constituting of an increase in MPM cases among the individuals comprised in the latter period [24]. However, the exact use of ICI in the selected cases was not specified by the authors, who only evaluated the differences between these two time intervals [24].

The limitations of the reported case, important when interpreting its findings, comprise the short follow-up interval taken into consideration, the selection bias, given the uniqueness of the clinical case, and the narrow statistical significance due to the inherently small sample size.

Therefore, it is equally crucial to maintain vigilant surveillance of the specific body region where the primary melanoma was initially diagnosed [8], but also of the whole cutaneous surface during subsequent dermatological and dermoscopic follow-up visits. Furthermore, we postulate that there might be numerous individuals unknowingly carrying genetic mutations associated with an elevated risk of melanoma, even though they may not be classified as high-risk patients from a clinical perspective, potentially evading early detection of melanoma.

## 4. Conclusions

In conclusion, this case report underscores the challenges in diagnosing an elevated number of primary melanomas within a single patient. Emphasizing the significance of immunohistochemistry and, notably, CDKN2A genetic testing, our findings highlight the essential role of these diagnostic tools in accurately discerning malignant melanocytic proliferations from nevi and in attaining a proper characterization of MPM cases.

## Figures and Tables

**Table 1:** Immuno-histochemical panel used for the first 4 diagnosed melanomas (year 2022).

Immuno-Histochemical Antibody	Clone	Manufacturer	Dilution	Host, Clonality
S100	15E2E2+4C4.3	BIOCARE, Pacheco, CA, USA	Ready-to-use (RTU) 7 mL	Mouse, Monoclonal
PAN-MELANOMA CK2	M2-7C10+M2-9E3+T31	BIOCARE	RTU 6 mL	Mouse, Monoclonal
p16	G175-405	BIOCARE	RTU 7 mL	Mouse, Monoclonal
Ki-67	SP6	BIOCARE	RTU 7 mL	Rabbit, Monoclonal
SOX-10	BC34	BIOCARE	RTU 7 mL	Mouse, Monoclonal
HMB-45	HMB45	BIOCARE	RTU 6 mL	Mouse, Monoclonal
CD8	SP16	BIOCARE	RTU 7 mL	Rabbit, Monoclonal

**Table 2:** Synthesis of primary cutaneous melanomas in the presented case.

Melanoma No.	Anatomic Site	Histopathological Subtype	Date of Diagnosis
1.	Left mammary gland (cutaneous)	Superficial spreading melanoma (SSM)	August 2022
2.	Left shoulder (cutaneous)	SSM	August 2022
3.	Right lateral cervical region	SSM	August 2022
4.	Left calf	SSM	August 2022
5.	Scalp	Nodular melanoma (NM)	February 2023
6.	Left lateral cervical region	NM	February 2023
7.	Mandibular region	NM	February 2023
8.	Right thigh	NM	August 2023
9.	Left foot	NM	August 2023

## Data Availability

The data generated in the present study are included in the figures of this article.
